# Feedback Design in Targeted Exercise Digital Biofeedback Systems for Home Rehabilitation: A Scoping Review

**DOI:** 10.3390/s20010181

**Published:** 2019-12-28

**Authors:** Louise Brennan, Enrique Dorronzoro Zubiete, Brian Caulfield

**Affiliations:** 1 Physiotherapy department, Beacon Hospital, Bracken Road, Sandyford Industrial Estate, Dublin 18, Ireland; 2 Insight Centre for Data Analytics, O’Brien Science Centre, University College Dublin, Dublin 4, Ireland; b.caulfield@ucd.ie; 3 School of Public Health, Physiotherapy and Sports Science, University College Dublin, Dublin 4, Ireland; 4 Department of Electronic Technology, Universidad de Sevilla, 41012 Seville, Spain; enriquedz@dte.us.es

**Keywords:** biofeedback, feedback, wearable sensors, rehabilitation, exercise, physiotherapy

## Abstract

Digital biofeedback systems (DBSs) are used in physical rehabilitation to improve outcomes by engaging and educating patients and have the potential to support patients while doing targeted exercises during home rehabilitation. The components of feedback (mode, content, frequency and timing) can influence motor learning and engagement in various ways. The feedback design used in DBSs for targeted exercise home rehabilitation, as well as the evidence underpinning the feedback and how it is evaluated, is not clearly known. To explore these concepts, we conducted a scoping review where an electronic search of PUBMED, PEDro and ACM digital libraries was conducted from January 2000 to July 2019. The main inclusion criteria included DBSs for targeted exercises, in a home rehabilitation setting, which have been tested on a clinical population. Nineteen papers were reviewed, detailing thirteen different DBSs. Feedback was mainly visual, concurrent and descriptive, frequently providing knowledge of results. Three systems provided clear rationale for the use of feedback. Four studies conducted specific evaluations of the feedback, and seven studies evaluated feedback in a less detailed or indirect manner. Future studies should describe in detail the feedback design in DBSs and consider a robust evaluation of the feedback element of the intervention to determine its efficacy.

## 1. Introduction

Biofeedback is a well-established technique in physical rehabilitation which aims to improve treatment outcomes by educating and engaging patients [1]. It involves providing an individual with additional information (feedback) on a physiological parameter, allowing the individual to influence the physiological parameter based on the feedback [2,3]. Biofeedback is a type of extrinsic feedback, meaning it is provided by forces external to the body, and is distinct from intrinsic feedback, which is information provided by an individual’s sensory systems. It can be categorised as mechanical, measuring body movement and forces, or physiological, measuring cardiovascular, respiratory or neurological parameters [4]. For the purposes of this review, the term ‘biofeedback’ will be used to describe the overall concept or technique, and ‘feedback’ will refer specifically to the information provided by the biofeedback system to the user.

A digital biofeedback system (DBS) used within physical rehabilitation consists of an input sensor, a data-processing system, and an output device which displays the feedback. DBSs have been shown to improve motor learning, engagement with and adherence to rehabilitation in musculoskeletal, neurological and orthopaedic populations [3,4,5]. They can improve awareness of exercise technique by providing information regarding movement that was previously unavailable. DBSs can generate objective measurements which act as rehabilitation goals, such as knee range of movement (ROM). With the appropriate data analytics and visualisations, the data collected can be compiled to create progress reports and allow remote monitoring by physiotherapists [6,7].

**Figure 1:**
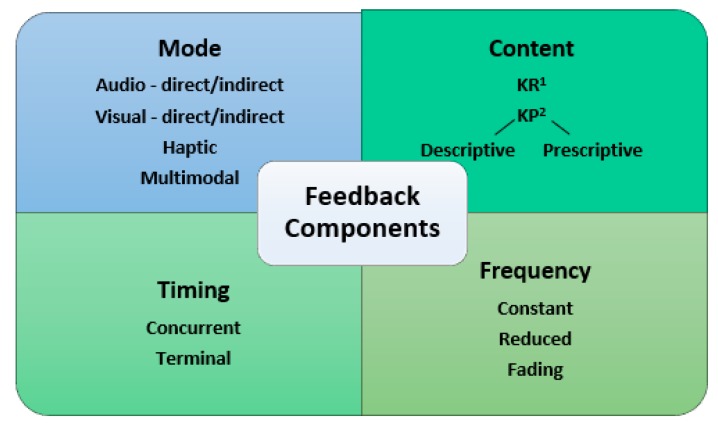
Components of feedback. KR ^1^ = knowledge of results; KP ^2^ = knowledge of performance.

Feedback consists of four main components (mode, content, frequency and timing), which each may be applied in a multitude of ways. Different combinations of these components will result in different feedback designs, and, theoretically, different effects on the user [8]. We have summarised the components of feedback that are observed in the relevant literature and presented them in Figure 1 as a framework within which to describe feedback interventions in detail. The first of these components is feedback mode, this may be visual, audio, haptic or multimodal (more than one mode represents the same variable at the same time). Through these modes, the feedback may be presented in a direct manner, e.g., a measure of ROM [9], or in an indirect manner, e.g., abstract graphical displays or gamified interfaces [10,11]. Feedback timing can be concurrent (also known as ‘real-time’ or ‘simultaneous’) or terminal, meaning it can be delivered during or after exercise execution, respectively. The frequency of feedback can be constant, reduced, or fading (decreasing over time). Feedback content may be knowledge of results (KR) or knowledge of performance (KP). In the context of rehabilitation, KR is information regarding the outcome of the movement, e.g., number of repetitions achieved, and KP is information regarding the execution of the movement, e.g., details of exercise technique [12]. A sub-category of KP feedback content is the quality of the feedback: is it descriptive (a description of errors) or prescriptive (a recommendation for correction of errors) [13].

As each component can uniquely influence motor learning and performance, the selection of appropriate components can be the difference between a DBS which improves outcomes and one which is redundant [5,8,14,15,16]. It is therefore important that researchers discuss the feedback components in their systems, and the rationale for selecting them, so that readers and users can fully understand the intervention and the evidence underpinning it. An alternative way of providing an evidence-based rationale for the choice of feedback components is to conduct an evaluation of the feedback within the context of the system. DBSs are complex interventions, comprising several elements which may impact the predicted outcome either individually or in combination [17]. For example, in addition to biofeedback, the rehabilitation outcome may be influenced by the chosen exercise programme, the educational material or the use of gamification. A methodology which includes a specific evaluation of the feedback components will provide information on the efficacy of the feedback and a justification for its inclusion, exclusion or modification in future iterations of the DBS.

A promising application of DBSs is in the field of unsupervised home rehabilitation. Home exercise programmes are a key component of physiotherapy-led home rehabilitation and are essential to build strength, improve function and reduce pain. These programmes largely consist of ‘targeted exercises’ (TEs), which we define as specific rehabilitation movements, prescribed in terms of ‘repetitions’ and ‘sets’ which are repeated and progressed over time, with the aim to improve strength, ROM or function in a specific joint, muscle or group of muscles. TE-based exercise programmes are frequently applied in early-stage post-operative rehabilitation [18,19,20] and in the management of many musculoskeletal disorders [21,22]. Additionally, TEs are prescribed individually to patients with a wide range of conditions including falls, chronic diseases and neurological conditions. In these instances, they are performed within holistic rehabilitation plans alongside task-orientated exercises and general physical activity. While TE-based programmes should be performed regularly at home or in another unsupervised setting, a wide variety of interrelating psychological, medical and environmental factors can act as barriers to full engagement in home rehabilitation [23,24]. Loss of interest due to the repetitive nature of the exercises can lead to poor adherence, and uncertainty regarding exercise technique can result in poor performance [25,26]. As the effectiveness of these exercises relies upon engagement with the prescribed protocol, novel solutions are required to encourage and facilitate performing TEs at home.

DBSs are relatively established in the specialties of gait assessment, balance re-education, incontinence rehabilitation and postural assessment; many examples of such systems can be found in the literature [27,28,29,30,31,32]. However, they are infrequently used alongside TEs in home rehabilitation. A summary of the use of feedback in DBSs for TEs does not exist, nor does guidelines for the design or evaluation of feedback in these kinds of systems. In order to produce recommendations to guide future DBS development, we must first understand the current use of feedback in DBSs for TEs, and the current practices of researchers with regards to rationalising and/or evaluating their feedback design choices.

This scoping review aims to address the need for a summary of the use of feedback in DBSs for TE-based home rehabilitation, by discussing how these DBSs employ feedback, what is the rationale for the choice of feedback components, whether feedback is being evaluated, and how it is being evaluated.

## 2. Materials and Methods

### 2.1. Search Strategy

We conducted a scoping review following guidelines provided by Arksey et al. [33] to answer the following research questions: how is biofeedback currently being applied in digital targeted-exercise home rehabilitation tools? Is the type of feedback used justified by rationale or evaluation? A systematic search strategy was used to identify relevant papers. The search was conducted in July 2018 and repeated in July 2019 to capture any newly published literature. We searched PubMed, Physiotherapy Evidence Database (PEDro) and Association for Computing Machinery (ACM) databases. Additionally, we checked the reference lists of included articles and carried out a search of grey literature through Google, Open Grey database (www.opengrey.eu) and Grey Literature Report (www.greylit.org). A review protocol was not published for this study. Search terms and strategies are detailed in Table 1.

Inclusion criteria for this review were as follows:

- Interventions: technology-based interventions where the user receives biofeedback. We will include only systems which have been evaluated on the target clinical population within its intended context of use.
- Exercise type: targeted exercises, i.e., specific rehabilitation movements which aim to improve strength, ROM or function in a specific joint, muscle or group of muscles. They are most often prescribed in terms of ‘repetitions’ and ‘sets’ and are intended to be repeated and progressed over time.
- Setting: the systems should be designed for use in an unsupervised home rehabilitation setting.
- Population: any clinical population receiving targeted exercises for rehabilitation.
- Studies written in the English language, from 2000 to July 2019.

Studies that focused on gait or balance were excluded, as these do not fall under the definition of targeted exercise. Additionally, reviews of biofeedback interventions already exist in these fields [27,30]. We excluded DBSs focusing on posture or trunk control, as this is typically treated with multi-faceted exercise programmes and not TEs. Research on exoskeletons and robotics was excluded as these are not designed as rehabilitation tools for unsupervised use outside of the clinical setting. Studies detailing systems which have not yet been tested on a clinical population were excluded, because systems in such an early stage of development may require significant changes before they can be tested on patients. A second reason for excluding such systems is that DBSs can only be evaluated in a valid manner on their target population. For example, patients use these systems differently than healthy individuals as they often know how to do a movement but are unable to do it due to pain or weakness. In this instance, a design flaw within a DBS which inadvertently encourages patients to go beyond their abilities, resulting in compensatory movement patterns, would only be identified in a patient cohort.

### 2.2. Study Selection

The articles identified through the search process were screened firstly by title and then by abstract if necessary. This process was split between two reviewers (LB and EDZ). Inter-rater reliability between researchers was calculated using a sample of 64 article titles, with a kappa coefficient of 1.0 indicating an excellent level of agreement. All papers selected for full review were assessed by both LB and EDZ, with any queries or discrepancies resolved by consensus. In incidences where two eligible papers discussed the same system, both papers were included as each contained different information, for example, a more detailed description of the technological aspects of the system or the evaluation process involved.

### 2.3. Data Extraction

A data-charting spreadsheet was created by LB and EDZ, with five main categories: population characteristics, exercise characteristics, technology comprising the system, feedback details and assessment of feedback. The ‘assessment of feedback’ category concerned any evaluation of feedback, ranging from quantitative analysis of feedback efficacy to qualitative data such as that from usability studies. Desired information was extracted from selected papers by LB and checked for accuracy by EDZ. In instances where two papers discussed the same system, data was collected on a ‘per paper’ basis, to be merged at a later stage.

### 2.4. Data Analysis

One reviewer (LB) went through all the extracted data to identify and analyse patterns, results and research gaps relevant to the objectives of this review. These insights were discussed with EDZ and BC.

## 3. Results

### 3.1. Search Results

**Figure 2:**
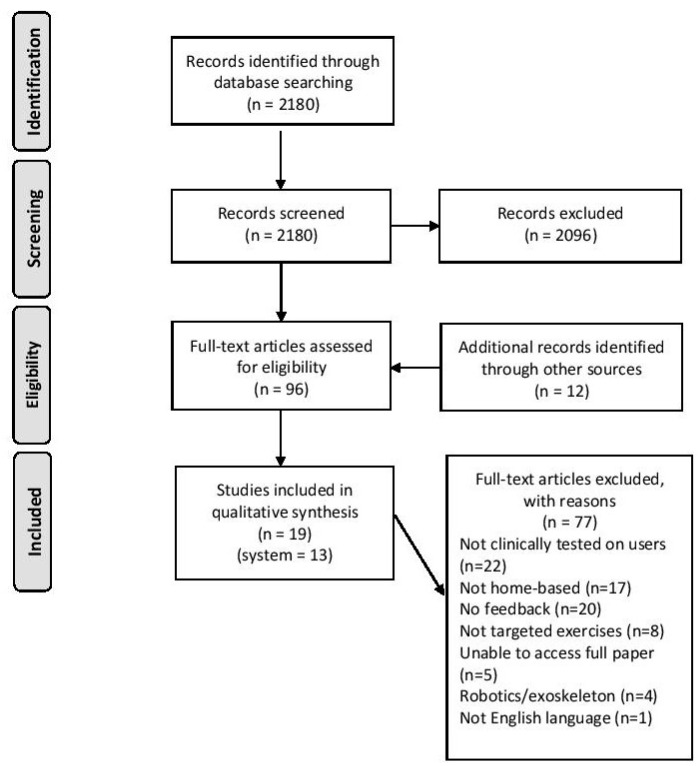
Flow diagram of the search strategy.

A total of 19 studies, describing 13 different biofeedback systems were identified for inclusion in this review. The original electronic database search in 2018 retrieved 1954 results, and the second search in 2019 retrieved a further 242 results. Between the two searches, PubMed provided 1081 results, PEDro 1033 and ACM 58. These 2196 results were screened by title and abstract, and 84 met the criteria for full-text review. A further 12 results were identified during the title/abstract/full-text review process through reference screening. After the full-text review of these 96 articles, 77 were excluded for the following reasons: not tested on users (n = 22), not designed for use at home (n = 17), did not contain biofeedback (n = 20), did not involve targeted exercises (n = 8), unable to access full paper (n = 5), intervention was robotics or exoskeletons (n = 4) and unavailable in English language (n = 1). The process is described via flow diagram in Figure 2. Nineteen articles remained after the screening process, describing 13 separate biofeedback home rehabilitation systems. Eight systems were described by one paper only [34,35,36,37,38,39,40,41], four systems were described by two separate papers [42,43,44,45,46,47,48,49] and one system was described by three papers [50,51,52]. All included papers fulfilled the inclusion criteria for this scoping review. In instances where multiple papers represent the same system, each paper contributed different information or additional details. For referencing purposes, when speaking of these systems, we will reference the paper which provides the most details; these ‘primary papers’ are indicated in Table 2.

### 3.2. System Characteristics

An overview of the general characteristics of each system is given in Table 2. Information is presented on a ‘per-system’ basis so that, where needed, information from included papers which describe the same system has been combined. This table describes the clinical context in which the system is designed to be used, the main hardware involved to receive sensor signals and deliver feedback, and how the feedback operates to give meaningful information to the user. Presentation of other system components, such as additional hardware, data processing software and motion analysis modules is beyond the scope of this review; full system details can be found in the referenced papers.

Five systems were developed for use in knee rehabilitation (chronic knee pain or post-operative care) [34,35,43,44,49], three for rehabilitation after a cerebrovascular accident (CVA/stroke) [38,46,51], two for elderly at risk of falls [37,43], and one each for rehabilitation related to carpal tunnel release surgery [36], total hip replacement [40], cerebral palsy [39] and chronic obstructive pulmonary disease (COPD) [41].

A wide range of input sensors was used across the systems: inertial measurement units (IMUs), smartphones, tablet computers, bend sensors, electrogoniometers, kinesthetic strain sensors, Kinect V2 and electromyography (EMG). Each system used only one type of input sensor, apart from Liu et al. [39] who used both EMG and accelerometers to collect exercise data. A narrower range of devices was used to provide feedback; these were: desktop or laptop computers (4); tablet computers (3); smartphone (3); television monitor (1) and in-garment light display (1).

The manner in which the feedback operates through interaction with the user and the exercise task is described in Table 2. Feedback details are described in greater depth in Section 3.3.

### 3.3. Feedback Components

Details of the feedback provided by each system are shown in Table 3. Feedback design components considered are mode, timing, content, the use of knowledge of results (KR) or knowledge of performance (KP), and whether the authors provided a rationale for their choice of feedback design. While a variety of visual, audio, haptic and multimodal modes could be used, all systems provided feedback of an audio or visual mode, and there was no haptic feedback. The BASE system by Doyle et al. [37] featured multimodal feedback, and five systems applied both audio and visual modes [35,38,39,40,44]. All systems featured concurrent, or ‘real-time’, feedback, and six of the systems also gave feedback with a time delay [35,38,40,41,43,44]. This combination of concurrent and delayed feedback manifested as real-time movement feedback via avatar, followed by a summary of the session or progress report. The terminal feedback was given after the completion of an exercise repetition, set or full session, and provided technique information or progress reports. Seven systems used descriptive feedback [34,36,37,39,40,43,46], three used both prescriptive and descriptive [35,38,51], one used prescriptive alone [41], and it was unclear what quality of feedback was used in two systems [44,49]. As each system provided feedback on more than one feature of the targeted exercise, KR and KP were noted to be applied in a variety of ways and eight systems applied both KP and KR [35,38,39,41,43,44,46,51]. Five systems used KR only [34,36,37,40,49] and no system used KP only. Constant feedback was given by all systems, but Durfee et al. [50] also employed a fading frequency protocol for some feedback features.

Three studies provided a rationale for the type of feedback used in the iteration of the system being evaluated in the study. Doyle et al. [37] incorporated multimodal feedback to compensate for potential sensory deficits in their target population of older adults and used concurrent feedback to assist in exercise completion. Accessibility was also considered by Giorgino et al. [46], who adapted their visual feedback so that bright colours were used only in essential feedback features, making it easier for cognitively impaired users to focus on these features. Carey et al. [52] applied a fading frequency KP feedback schedule, based upon the principle that ‘excessive extrinsic feedback interferes with one’s own intrinsic error detection capability, which can disrupt learning’ [53], and a constant KR schedule was used to maintain motivation levels during exercise. Two of the systems which did not state the rationale for using feedback were based upon a rehabilitation game [39,40]. In both cases, the authors discussed the benefits of rehabilitation games, stating that these benefits were fundamental to the system design. We identified these studies in Table 3 to clarify that, while no explicit rationale was given for feedback type, a process of evidence-based game design guided the feedback development.

### 3.4. Feedback of Exercise Components

**Figure 3:**
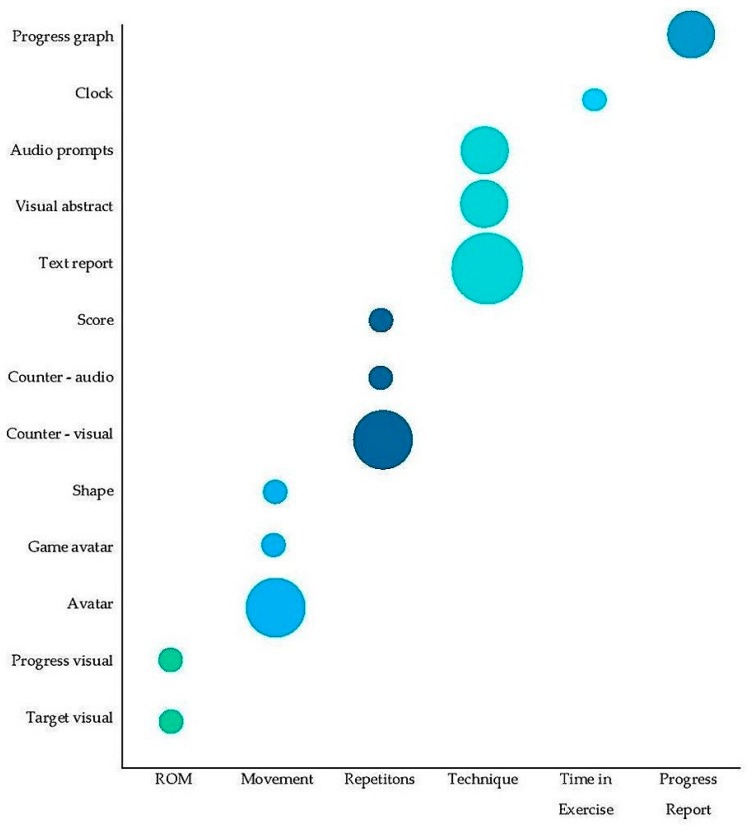
Feedback features used to represent exercise components. The size of the circle corresponds to frequency that each feedback feature was used to represent the exercise component.

Six main components of exercise were represented in the feedback; these are: ROM, movement (a ‘mirroring’ of user’s movements), repetitions, technique, time spent exercising and reports of progress with, or adherence to, an exercise programme. Exercise components were at times represented in a concrete design style, for example realistic human avatars [35,37,38,39,41], repetition counters [35,37,41,43,46] and technique feedback via text [35,38,41,49,50]. These designs can be contrasted with abstract avatars [39,43], scores to indicate repetitions [39,40] and colours [43], faces [46] or game features [39] to comment on technique. Figure 3 shows how each exercise component was represented through feedback, and which features were most commonly employed.

### 3.5. Evaluation of Feedback

Eleven of the 19 papers included in this review conducted some aspect of evaluation of the feedback used in their system (see Table 4). We determined this by selecting all the papers which reported, whether in detail or briefly, upon the feedback design in the results section. The other eight studies focused on general system results, for example, clinical efficacy, and did not provide evaluation data specifically regarding feedback. Table 4 details the study design, participant characteristics, methodology and outcome measures used in the evaluation studies. Only results related to feedback are presented in this section, and they are grouped by the methodology employed to evaluate the feedback.

Four studies conducted detailed evaluations of feedback within their systems. Both studies by Ayoade et al. [42,43] compared the use of the falls and/or knee rehabilitation feedback systems with standard care (exercise booklets). Outcomes related to feedback were measured by observations and interviews. Participants reported that, in comparison to standard care, the feedback allowed them to focus on their own movements and to achieve better performance. Multiple feedback features were appealing to users, including the avatar, ROM graphic and progress reports. In focus groups, Doyle et al. [37] tested four types of visual feedback and two types of audio feedback. Users unanimously chose a high-fidelity ‘matchstick man’ as their first preference of a graphic to represent themselves on the screen while exercising; they report that it was ‘very visible’ and ‘a good clear demonstration’. For repetition counting, 80% of users preferred a ‘ding’ sound to a count of ‘one, two, three...’, which was felt to be distracting. Carey et al. [52] assessed the efficacy of the tracking feedback application for hand rehabilitation exercises after stroke by conducting a randomised controlled trial. An intervention group (n = 10) trained with the tracking feedback, while a control group trained with the application minus the tracking feedback feature. The intervention group improved significantly in all behavioural outcome measures, while the control group showed significant improvements in only one test. However, fMRI studies did not find a consistent pattern of brain reorganisation in either group. Efficacy of audio feedback to improve exercise technique was investigated by Spina et al. [41]. Seven participants with COPD used the COPD Trainer system to complete a targeted exercise programme, and the system provided corrective audio feedback each time it detected an erroneous exercise repetition. Across 354 incidences of audio feedback, 297 were followed by a correctly executed repetition, and 57 were ‘ignored’, resulting in subsequent erroneous repetitions.

Several studies investigated feedback with users through exploratory, qualitative means. Ananthanarayan et al. [34] and Argent et al. [35] applied the Think Aloud protocol and semi-structured interviews in their usability sessions, which revealed both positive and constructive insights from users. Participants using Ananthanarayan et al.’s PtVIZ system reported that the electroluminescent wire bar graph was intuitive and helpful but did not give sufficient feedback for those making slow progress. Argent et al. provide direct quotes from users regarding the feedback design: ‘It made what are otherwise boring exercises more interesting’; ‘On the graph I don’t know what the interpretation is supposed to be’. Participants also requested additional desired feedback features, such as a measure of ROM or elements of gamification. Ling et al. [40] gathered positive and negative comments from both patients and physiotherapists concerning the interactions between feedback, game design, exercise task and the user. Comments included: ‘It is a very useful game for balance training, and it reacts very well to the movements of the player.’; ‘It is difficult for me to pay attention to the arrows indicating which leg I should use.’ The authors included a list of the implications these comments would have on future prototype iterations.

The final evaluation method was the use of close-ended questionnaires. Physiotherapists in Ling et al.’s study answered a quantitative questionnaire, including a question regarding appropriateness of the feedback to ‘encourage adherence to the game and keep track of the patient’s recovery status’: on a scale of −3 to +3, all games were rated between 1 and 3. Giorgino et al. [46] administered a user satisfaction survey to 13 participants, which addressed feedback in an indirect manner. Questions included: ‘Do you easily understand the screen?’ (Agree = 2, Neutral = 7, Disagree = 4); ‘Are the exercises easy to understand?’ (Agree = 9, Neutral = 3, Disagree = 1); and ‘Do you get bored?’ (Agree = 2, Neutral = 4, Disagree = 7). Durfee et al. [50] also administered a usability survey to participants (n = 19), which contained one question specifically related to feedback. Participants used a five-point Likert scale to respond to the statement “The scores and comments I received after each trial helped me” as follows: disagree 3, no opinion 1, agree 14, strongly agree 1. A questionnaire given by Liu et al. [39] to 20 child participants and their parents included the statement ‘The feedback information of score, sound and graphic help me to finish the games better’. This statement scored 3.75 ± 0.72 on a scale of 1 (‘don’t want to play’) to 5 (‘tried to get higher score’). Seventy percent of participants reported that they liked the feedback design (sounds and graphics).

## 4. Discussion

### 4.1. Systems Identified

The aim of this review was to identify and discuss the feedback used in DBSs available for targeted exercise rehabilitation in the home. Our search found 13 systems have been developed since the year 2000, which appears to be a low number, considering the importance of TEs in Physiotherapy rehabilitation and the advances in technology over the last two decades. A multitude of research studies exists surrounding the creation of digital systems for targeted exercise detection and classification using a variety of motion sensors [32,54,55,56,57,58]. Challenges to creating such systems are noted in this body of literature and may explain why so few systems were identified. These include the selection of an appropriate input sensor, accurate and valid movement analysis of TEs, and the identification of suitable clinical contexts. To overcome these challenges, certain trends were noticed in the identified systems. Considering the question of which input sensor to use, several methods of motion analysis currently applied in biofeedback, such as electromyography, real-time ultrasound and marker-based motion tracking systems are costly and require supervision to set up and operate [59,60,61]. In contrast, IMUs are small, relatively inexpensive and easy to use, making them ideal for use in a home rehabilitation setting, while also capable of delivering accurate and valid analysis of TE data [56,62,63]. We found that five of the included systems used IMUs, and, in addition, one used the IMU within a smartphone and one unspecified ‘movement sensor’ likely referred to an IMU. The trend towards using IMUs as input sensors indicates that they solve several of the usability challenges in home rehabilitation systems.

A further trend noticed was a commonality in clinical context, with five systems designed for use in knee joint rehabilitation. An increasing incidence of total knee replacement in many countries is creating a burden on healthcare systems [64]. Several of the papers [35,44,49] state that the need for innovative models of care to promote self-management and reduce costs was a primary motivator behind the development of their systems. Aside from being a suitable clinical context, rehabilitation of the knee joint has practical and biomechanical advantages for biofeedback systems. Early-stage post-operative knee rehabilitation protocols typically involve performing a small set of widely-recommended targeted exercises [18], which allows for a comprehensive prototype to be developed and tested widely. This is ideal for systems with motion analysis, where accuracy is reduced in circumstances of excessive unseen data or data variance. Additionally, relatively simple hinge joints, such as the knee or elbow, allow for relatively straightforward data collection and feedback, compared to a joint with greater degrees of freedom, or a multi-joint movement analysis system. Another compelling clinical context for the development of DBSs is in stroke rehabilitation; three of the included systems were designed for this context. Motor recovery after a stroke often requires extensive, repetitive training of both TEs and functional movements. Digital feedback systems, with or without gamification, can assist in maintaining engagement while preventing the development of compensatory movement patterns by providing accurate feedback [65,66]. Promoting engagement also allows users to reach the critical volume of training required for muscle hypertrophy, a common rehabilitation goal across many contexts [67].

Two systems provided feedback on TEs in a game format. Liu et al. [39] delivered feedback only during the gameplay, while the ‘Fietsgame’ system by Ling et al. [40] also provided delayed KR in a platform which can be accessed by patient and therapist. This structure of game feedback supported by a platform containing delayed feedback is commonly seen in serious games, for example, the KineActiv system by Muñoz et al. [68] stores information on repetition accuracy and duration in a cloud database.

### 4.2. Feedback Components

Visual feedback, which was used in all the included systems, can improve exercise performance in home rehabilitation by two methods. Firstly, visualisations can enhance engagement and distract from the exercise task; participants in Ling et al. report that ‘After you get used to playing the exercise games, you have a lot of fun’. Secondly, visualisations can communicate concepts such as ROM or exercise technique in a more clear and objective manner. Ayoade et al. quote one participant discussing their ROM graphic: “It is very useful to have fullness of each exercise calibrated by colour code” [42]. To not overwhelm the user with visual information, feedback should be provided only on the most important features of the task [8], and other systems have employed multi-modal feedback to improve accessibility especially in populations which may experience sensory loss [10,69].

As many of the included systems provide real-time feedback, the information should be intuitive and easy to interpret. Most concurrent feedback was given visually, as this is a natural and intuitive way of receiving information for many people. However, while concurrent visual feedback can improve motor performance initially, these improvements are often not maintained on follow-up assessment [70,71]. This may be explained by users becoming reliant on the constant feedback and therefore losing awareness of their own intrinsic feedback (i.e., proprioception). DBSs can aim to counteract this reliance by using a fading-frequency feedback protocol, or using ‘bandwidth feedback’ which delivers feedback when movement errors exceed a threshold level which is appropriate for the user’s stage of learning [72,73]. Audio feedback is likely to be less intuitive but can deliver a high concentration of information through the use of various dimensions of sound, such as volume, pitch and timbre.

### 4.3. Rationale for Feedback

Only three papers provided a rationale for the type of feedback used within their systems [37,46,50]. This could be seen as a missed opportunity by the majority of the included papers to discuss the detailed evidence behind how the feedback system was designed to influence motor learning, engagement and accessibility in rehabilitation. Alternatively, this finding may simply reflect the fact that the focus of many papers was not specifically on the granular details of feedback designs, but on the overall system development and evaluation. In this case, the discussion of feedback may not have been a priority for the authors. The included papers described the evidence behind how other aspects of the systems were designed to meet the goals of motor learning, engagement and accessibility.

### 4.4. Feedback Evaluation

Ten papers (eight systems) conducted evaluations of their system, which included an evaluation of the feedback itself in some capacity. While this review found that evidence-based rationale is not usually given to justify the feedback design, these evaluations are a prospective method of generating an evidence-base. Evaluations varied from a detailed comparison of different feedback designs to questionnaires on a whole-systems level which addressed feedback in a minor or indirect way. Where the clinical efficacy was assessed, the study design was not specific enough to determine the effect of the feedback, as separate from the effect of the whole system [39,40,43]. The exception to this is Carey et al. [52], which was the only study to conduct a randomised controlled trial to compare the effects of the system with feedback versus without feedback. At times, the usability evaluations were insufficiently detailed. For example, Durfee et al. [50] received largely positive feedback to the statement ‘The score and comments I received after each trial helped me’, but through the Likert scale design they were unable to uncover why three participants (15% of respondents) chose to disagree with this statement. A qualitative approach during usability testing, as seen in several of the included systems [34,35,37,39,43] can reveal helpful insights and allow for a user-centred design approach to biofeedback systems evaluation.

### 4.5. Review Limitations

This review has several limitations. No analysis of the quality of the papers was conducted, as this scoping review instead aimed to map the literature in this field and answer the main research questions posed in the introduction. Relevant studies may have been missed if they were published outside of the English language, outside of the time frame, in a different database or were not captured by the search strategy. Reporting of feedback components within the included papers was often not thorough, particularly in cases where the authors’ main focus was not on biofeedback. Therefore, some details we sought may be missing or not reported in detail.

### 4.6. Recommendations for Future Research

Future research should focus on the development of DBSs through robust design methodologies that combine evidence-based feedback interventions with qualitative user-centred design processes. It is recommended that future studies should describe feedback design and rationale for its selection in greater detail, to allow clinicians and researchers to fully understand the intervention and its intended effects. Evaluation of feedback as well as an overall system evaluation will help identify both independent effects of feedback and the overall efficacy of the entire intervention as an adjunct to physiotherapy treatment.

## 5. Conclusions

DBSs can provide support to patients as an adjunct to physiotherapy-led home rehabilitation. The feedback components chosen in the system development can influence motor learning and user engagement. DBSs designed for TEs in home rehabilitation utilise a variety of input sensors, with IMUs being most commonly used, and tablets, smartphones or laptops providing the majority of feedback. Feedback was characterised by being mainly visual, concurrent and descriptive, with KR more commonly featured than KP. Authors reported the overall system design in detail but rarely reported a rationale for the choice of feedback. Few studies evaluating feedback efficacy on its own, and feedback was often evaluated as part of the whole system. Future studies should provide more details on feedback interventions, and apply more focused evaluation methodologies to determine the value of feedback within the overall DBS.

## Figures and Tables

**Table 1:** Search strategy.

Database	Search Strategy
PubMed	((mobile app* OR mhealth OR mobile health OR ehealth OR smartphone OR acceleromet* OR wearable OR sensor system OR sensor-based system OR IMU OR inertial measurement unit* OR internet) AND (biofeedback OR bio-feedback OR feedback) AND (rehabilitation OR physiotherapy* OR physical therap*))
ACM	(+ (web internet “mobile app*” mhealth “mobile health” ehealth smartphone acceleromet* wearable “sensor system” “sensor-based system” IMU “inertial measurement unit*”) + (biofeedback “bio-feedback” feedback) + (rehabilitation physiotherapy* “physical therapist” “home exercise”))
PEDro	Biofeedback ‘Feedback technology’ Mhealth ‘Technology rehabilitation’ ‘Mobile rehabilitation’ ‘Mobile exercise’ ‘Wearable’ ‘Sensor exercise’

**Table 2:** Characteristics of the systems.

Ref	Clinical Context	System Components	Feedback Design
Ananthanarayan et al., 2013 [34] ‘PT Viz’	Condition: Chronic knee pain/post knee surgery Exercise: knee flexion and extension	Input sensor: neoprene bend sensor at back of knee, held in place by neoprene sleeves around thigh and calf Feedback device: electroluminescent wire lights in thigh sleeve	As user bends the knee, bars of electroluminescent wire light up; fully lit bars indicate full knee bend.
Argent et al., 2019 [35]	Condition: TKR ^1^ or UKR ^2^ Exercise: post-operative knee ROM and strengthening	Input sensor: IMU ^3^ in sleeve around calfFeedback device: tablet with application	Tablet application displays a 3D human avatar mirroring user’s lower limb movement. Repetitions are indicated with beeping noise and on-screen counter. A text report provides technique feedback.
Ayoade et al., 2013 [42], 2014 * [43]	Condition: TKR; falls Exercise: post-operative knee; falls rehabilitation	Input sensor: IMU (two for knee module, six for falls) Feedback device: computer with visualisation software	A stick-figure avatar simulates lower limb (knee) or body (falls) movements. The knee module contains a coloured fan graphic to indicate ROM progress, with corresponding colours indicating ROM per-repetition and a weekly progress chart.
Blanquero et al., 2019 [36] ‘ReHand’	Condition: carpal tunnel release Exercise: fingers & wrist mobility dexterity, co-ordination	Input sensor: tablet touch screen Feedback device: tablet with Android application	The user performs exercises by touching the screen. Application displays exercise instructions and circles on which to place fingertips. Circles move with fingers, providing feedback on direction of movement and proximity to target. A countdown clock appears on screen.
Correia et al., 2018 * [44], 2019 [45]	Condition: TKR Exercise: post-operative knee ROM ^4^ and strengthening	Input sensor: IMU (3: calf, thigh, chest) Feedback device: tablet with application	The user aims to fill a ROM progress bar, earning stars by surpassing the target ROM. Movement or posture errors are communicated with audio and video feedback. A simple human avatar displaying user’s posture, a repetition counter and a timer also appear on screen.
Doyle et al., 2010 [37] ‘BASE’	Condition: older adults at risk of falls Exercise: Otago programme for strength & balance	Input sensor: IMU (2), webcam/tracking markers (3) Feedback device: laptop with application	An avatar simulating user’s movements is superimposed with a ROM target line. Repetitions are counted on screen as the lower limbs passes this line. Walking exercises utilise audio prompts for feedback. Weekly progress record reports on compliance, target acquisition and repetition counts.
Durfee et al., 2009 * [50], Durfee et al. 2009 [51], Carey et al. 2007 [52]	Condition: CVA ^5^ Exercise: wrist & finger extension	Input sensor: electrogoniometer Microcontroller interface box Feedback device: laptop with application	Joint motions control the movement of a ball on screen. The user must trace a variety of waveform patterns with the ball. The resulting trace provides accuracy feedback, as does a text-based technique report and accuracy score.
Giorgino et al., 2009 * [46], 2009 [47] ‘NR System’	Condition: CVA Exercise: upper limb simple movements; eating & combing	Input sensor: garment with kinesthetic strains sensors Feedback device: computer with application	Motion recognition software is trained by user performing exercises under supervision. User then exercises independently, and computer displays repetition counter and smiling/frowning faces indicating repetition classification.
Lin et al., 2018 [38]	Condition: CVA Exercise: upper limb simple movements	Input sensor: IMU (2: upper arm, forearm) Feedback device: smartphone with application	Smartphone application displays a human avatar simulating movement in front or side views. After six repetitions, system provides auditory and visual technique feedback and prompts.
Ling et al., 2017 [40] ‘Fietsgame’	Condition: THR ^6^ Exercise: lower limb, e.g., steps, squats, lunges	Input sensor: Microsoft Kinect V2 Feedback device: television monitor	A human avatar simulating user’s movement performs a programme of games (selection of six for exercises, and six for balance training). Gamification feedback elements include scores, awards and sounds. Additional feedback on results and performance from game-specific features, e.g., background avatars dancing/clap if exercise performed correctly.
Liu et al., 2017 [39]	Condition: cerebral palsy Exercise: upper limb simple movements	Input sensor: surface EMG ^7^ circuit, accelerometer Feedback device: tablet with application	Upper limb joint motion & muscle activity signals control three different games. Gaming-style avatars (bird, cat, magician) complete tasks with gamified audio/visual elements, scores, performance grading, mean absolute value.
Smittenaar et al., 2017 * [49], Mecklenburg 2018 [48]	Condition: chronic knee pain Exercise: knee ROM and strengthening	Input sensor: motion sensors (2: thigh and calf) Feedback device: smartphone with application	Android platform delivers real-time technique feedback and progress screen.
Spina et al., 2013 [41] ‘COPD ^8^ Trainer’	Condition: COPD Exercise: upper and lower limb variety	Input sensor: smartphone (IMU) in holster (relocated throughout exercising) Feedback device: smartphone with application	Application features real-time audio error correction (e.g., ‘move slower’) and repetition counting. A performance summary appears after exercising.

* Primary paper where two papers discuss one system. ^1^ TKR = Total Knee Replacement; ^2^ UKR = Unicompartmental Knee Replacement; ^3^ IMU = Inertial Measurement Unit; ^4^ ROM = Range of Movement; ^5^ CVA = Cerebrovascular Accident; ^6^ THR = Total Hip Replacement; ^7^ EMG = Electromyography; ^8^ COPD = Chronic Obstructive Pulmonary Disease.

**Table 3:** Feedback components.

Name	Mode	Timing	Content	Quality	Rationale for Type of FB
Ananthanarayan et al., 2013 [34]	Visual	Concurrent	KR ^1^	Descriptive	Not stated
Argent et al., 2019 [35]	Visual & audio	Concurrent & delayed	KR & KP ^2^	Descriptive & prescriptive	Not stated
Ayoade et al., 2013, 201 [42,43]	Visual	Concurrent & delayed	KR & KP	Descriptive	Not stated
Blanquero et al., 2019 [36]	Visual	Concurrent	KR	Descriptive	Not stated
Correia et al., 2018 [44]	Visual & audio	Concurrent & delayed	KR & KP	Unclear	Not stated
Doyle et al., 2010 [37]	Multimodal	Concurrent	KR	Descriptive	Multimodal feedback to compensate for sensory impairments. Real-time feedback to assist exercise completion. User preference dictated choice of audio and visual feedback style.
Durfee et al., 2009 [50], Durfee et al. 2009 [51], Carey et al., 2007 [52]	Visual	Concurrent & delayed	KR & KP	Descriptive & prescriptive	Faded frequency KP used to prevent excessive extrinsic feedback interfering with user’s intrinsic error detection capability. Constant KR used to maintain motivation levels. State that tracking training emphasises motor learning principles outlined in Schmidt et al. [53]
Giorgino et al., 2009 [46], 2009 [47]	Visual	Concurrent	KR & KP	Descriptive	Visual feedback adapted for cognitively impaired users.
Lin et al., 2018 [38]	Visual &audio	Concurrent & delayed	KR & KP	Descriptive & prescriptive	Not stated
Ling et al., 2017 [40]	Visual & audio	Concurrent & delayed	KR	Descriptive	Not stated (Game)
Liu et al., 2017 [39]	Visual & audio	Concurrent	KR & KP	Descriptive	Not stated (Game)
Mecklenburg et al., 2018 [48], Smittenaar et al., 2017 [49]	Visual	Concurrent	KR	Unclear	Not stated
Spina et al., 2013 [41]	Audio & Visual	Concurrent & delayed	KR & KP	Prescriptive	Not stated

^1^ KR = Knowledge of Results; ^2^ KP = Knowledge of Performance.

**Table 4:** Evaluation of feedback.

Name	Study Design	Participant Characteristics	Methodology	Outcome Measures
Ananthanarayan et al., 2013 [34]	Usability case series	N ^1^ = 6 Sex = four females, two males Age = 20–37 Country = USA Inclusion = history of knee surgery (n = 4) or chronic knee pain (n = 2)	Background questionnaire and usability session, followed by semi-structured interview.	Think aloud protocol & semi-structured interviews.
Argent et al., 2019 [35]	Usability case series	N = 15 Sex = nine females, six males Age = 63 ± 8.32 years Country = Ireland Inclusion = recent history of TKR ^2^ or UKR ^3^	Participants used system at home for two weeks, then completed outcome measures. The first group (n = 5) were recruited at the end of their acute rehabilitation, the second group were recruited prior to surgery and used the system throughout their rehabilitation experience.	US ^4^, uMARS ^5^, and semi-structured interview.
Ayoade et al., 2013 [42]	Within-subjects systems comparison study	N = 11 (falls n = 5, TKR = 6) Sex = three females, eight males Age = 60 years and above Country = Scotland Inclusion = >60 years, history of falls or history of knee replacement	Evaluation of both the knee and falls systems consisted of two single-session assessments: a lab-based usability study (n = 5) and a home-based systems comparison study (n = 6). In the home-based study, participants first completed the exercises using booklets, then using the feedback system.	Observations, repetition pace, questionnaires, and semi-structured interviews.
Ayoade et al., 2014 [43]	Randomised controlled trial	N = 21 Sex = 11 females, 10 males Age = 47–85 years Country = Scotland Inclusion = undergoing TKR surgery	Participants randomised into rehabilitation visualisation system group, who used the feedback system at home, and control group, who received standard care of exercise DVD and booklet. Duration: 6 weeks.	Knee ROM ^6^, Oxford Knee Score, Intrinsic Motivation Inventory, adherence questionnaire, and SUS.
Doyle et al., 2010 [37]	Usability focus groups and case series	N = 12 Sex = not stated Age = older adults Country = Ireland Inclusion = older adults	First usability session: participants performed exercises with system using each of four different types of visual feedback, then completed walking exercises to evaluate two types of audio feedback. Second usability session: participants used system at home, completed system-navigation tasks.	Think Aloud protocol, observations, and interviews.
Carey et al., 2007 [52]	Randomised controlled trial	N = 20 Sex = five females, 15 males Age = 66.65 ± 9.6 years Country = USA Inclusion = chronic CVA ^7^, 30–80 years, visually able to use system, minimum ROM criteria applied	Intervention group (n = 10) used full system including tracking feedback at home, control group used system without tracking feedback function. Completed 180 trials per day for 10 days.	Battery of clinical hand assessments-Box and Block, Jebsen Taylor, finger ROM, and finger tracking activation paradigm using fMRI ^8^
Durfee et al., 2009 [50]	Usability study	N = 20 Sex = five females, 15 males Age = 66.65 ± 9.6 years Country = USA Inclusion = chronic CVA, 30–80 years, visually able to use system, minimum ROM criteria applied	Participants completed RCT ^9^ as described in Carey et al., above. Then answered usability survey via telephone.	ix-question Likert scale questionnaire.
Giorgino et al. 2009 [47]	Usability study	N = 13 Sex = four females, nine males Age = 32–79 (mean 50) years Country = Italy Inclusion = hemiplegia & mild motor/cognitive impairment post CVA	Participants used system and completed evaluation questionnaire (limited details available).	User satisfaction survey.
Ling et al., 2017 [40]	Pilot usability study	N = 9 (two physiotherapists, seven patients) Sex = six females, three males Age= 74.5 ± 8.3 years (patients) Country = Netherlands Inclusion = post hip joint replacement of hip hemi-arthroplasty	Patient participants played six games under the guidance of a physiotherapist during a 60 min session. All participants completed outcome measures afterwards.	elf-report questionnaires, ‘general feedback’, objective data from software, e.g., knee angle and step width.
Liu et al., 2017 [39]	i. Usability testing. ii. Intervention case series.	N = 20 Sex = 12 females, eight males Age = 8.7 ± 2.8 years Country = China Inclusion = children with cerebral palsy diagnosis, voluntary movement and ‘normal cognitive capacity’	i. ‘Game experience testing’: participants (n = 20) played each game in controlled environment ii. ‘Training effect test’: participants (n = 3) completed game training 2–3 times a week for one month, followed by once a week for 1.5 months.	i. Questionnaire, training time. ii. Fugl-Meyer Assessment & ADL ^10^ scale for upper extremity. SEMG ^11^ force and game accuracy.
Spina et al., 2013 [41]	Pilot case series study	N = 7 Sex = four females, three males Age = 60 ± 10 years Country = Netherlands Inclusion = COPD ^12^, undergoing pulmonary rehabilitation	In controlled environment, participants received instructions and systems was set up during ‘teach mode’. Participants then independently completed three sets of ten repetitions of each exercise.	ystem accuracy Impact of audio feedback on performance.

^1^ N = Total Sample; ^2^ TKR = Total Knee Replacement; ^3^ UKR = Unicompartmental Knee Replacement; ^4^ SUS = Systems Usability Scale; ^5^ uMARS = user version of the Mobile Application Rating Scale; ^6^ ROM = Range of Movement; ^7^ CVA = Cerebrovascular Accident; ^8^ fMRI = Functional Magnetic Resonance Imaging; ^9^ RCT = Randomised Controlled Trial; ^10^ ADL = Activities of Daily Living; ^11^ SEMG = Surface Electromygraphy; ^12^ COPD = Chronic Obstructive Pulmonary Disease.
